# Influence of Nonenzymatic Posttranslational Modifications on Constitution, Oligomerization and Receptor Binding of S100A12

**DOI:** 10.1371/journal.pone.0113418

**Published:** 2014-11-26

**Authors:** Kerstin Augner, Jutta Eichler, Wolfgang Utz, Monika Pischetsrieder

**Affiliations:** 1 Department of Chemistry and Pharmacy, Food Chemistry Division, Friedrich-Alexander-Universität Erlangen-Nürnberg (FAU), Erlangen, Germany; 2 Department of Chemistry and Pharmacy, Medicinal Chemistry Division, Friedrich-Alexander-Universität Erlangen-Nürnberg (FAU), Erlangen, Germany; University of Miami, United States of America

## Abstract

This study examined the effect of methylglyoxal (MGO)-derived nonenzymatic posttranslational modifications (nePTMs) on the binding affinity of S100A12 to its natural receptor for advanced glycation end-products (RAGE). Binding of MGO-modified S100A12 to RAGE decreased significantly with increasing MGO concentration and incubation time. Ca^2+^-induced S100A12 hexamerization was impaired only at higher MGO concentrations indicating that the loss of affinity is not predominantly caused by disturbance of ligand oligomerization. nePTM mapping showed carboxyethylation of lysine (CEL) and the N-terminus without preferential modification sites. Besides, hydroimidazolone, hemiaminals, argpyrimidine, and tetrahydropyrimidine rapidly formed at R21. Even at the highest modification rate, hexamerization of synthesized CEL-S100A12 was unaffected and RAGE-binding only slightly impaired. Thus, nePTMs at R21 seem to be the major cause of MGO-induced impairment of S100A12 oligomerization and RAGE binding.

## Introduction

Nonenzymatic posttranslational modifications (nePTMs) of proteins are formed by spontaneous chemical reactions of amino acid side chains by diverse mechanisms such as oxidation or glycation. Protein glycation results from the reaction between reducing sugars and amino groups of proteins. Advanced glycation end-products (AGEs) are formed either by degradation of primary glycation products or by the reaction of amino acid side chains with reactive carbonyl compounds such as glyoxal, methylglyoxal (MGO), or 3-deoxyglucosone [1], [2]. Increased levels of glycation products, AGEs and/or oxidation products have been associated with various diseases such as diabetes [3], uremia [4], or chronic liver disease [5].

nePTMs severely alter the proteins’ primary structure by diverse covalent modifications of amino acid side chains. Consequently, modifications of the protein structure may lead to a conformational change resulting in a change of biological function. Alternatively, nePTMs may be located directly in protein–protein-, enzyme–substrate-, or protein–DNA interaction sites. Thus it was shown, for example, that the glycation of aldehyde reductase impaired enzymatic activity, but it is not clear which glycation products and which sites were responsible for this loss of activity [6]. The reaction of human serum albumin with the AGE-precursor MGO led to a loss of esterase activity. Because R410, which was predominantly modified, is located in the active site, it was concluded that the AGE formation directly disrupted the enzyme–substrate interaction [7]. Finally, intermolecular cross-linking of proteins may drastically change protein properties and impair their biological functionality. Cross-linking of collagen, for example, increases the stiffness of the collagen network, thus decreasing the resistance of cartilage to fatigue [8].

The present study investigated the influence of nePTMs on the ligand–receptor interaction of the protein S100A12 and the receptor for advanced glycation end-products (RAGE). RAGE belongs to the immunoglobulin superfamily of cell surface receptors. Several endogenous ligands of RAGE have been described, such as amphoterin (HMGB1) or S100 proteins. RAGE is expressed at relatively low levels in most cell types and tissues, but its expression is upregulated at sites where its ligands accumulate. Because most of these ligands are related to various diseases, the engagement of RAGE triggers diverse signaling cascades and activates transcription factor nuclear factor-κB (NF-κB) leading to pro-inflammatory reactions. Thus, RAGE is associated with pathologies like chronic inflammation, atherosclerosis, neurodegenerative diseases, diabetic complications, and cancer [9]. The natural RAGE ligand S100A12 is involved in the inflammatory response by triggering cell activation and generation of pro-inflammatory mediators via RAGE [10]. S100A12 is a member of the S100 protein family, a family of small (10–12 kDa), acidic, Ca^2+^-binding EF hand proteins [11]. Like most S100 proteins, S100A12 forms homodimers, but hexamerizes at elevated Ca^2+^-concentration by the association of three dimers [12]. The hexamer is the active form of S100A12, which binds to RAGE dependent on Ca^2+^- and Zn^2+^-ions [13].

It is well established that RAGE signaling is strongly modulated by the presence of nePTMs [14]. The introduction of AGEs, such as *N^ε^*-carboxymethyllysine (CML) or *N^ε^*-carboxyethyllysine (CEL), triggers the binding of non-natural ligands, e.g. human serum albumin, to RAGE [15]. It was proposed that AGE formation of non-ligands generates novel binding sites and that, for example, the negatively charged CEL fits inside a positively charged cavity of the V-domain of RAGE [16]. Apart from that, it can be expected that nePTM formation may also impair binding of RAGE to its endogenous ligands, such as S100A12. S100A12 is expressed at inflammation sites and nePTM formation is promoted under inflammatory conditions [17], [18]. Although the biological half-life of peptide ligands is relatively short, CML-modified S100A8 and S100A8/9 have been detected in inflamed gut tissue [19]. Consequently, it was postulated that CML modifications may enhance S100-induced inflammatory reactions. However, CML is not the most prevalent nePTM *in vivo*. In diabetic patients, for example, MGO-derived hydroimidazolone was detected in 20–40 times higher concentrations than CML. MGO reacts rapidly with amino acid side chains so that structural and functional effects can already be observed after a few hours of reaction time [2]. Thus, MGO may also severely modify protein ligands with a relatively short half-life. Because it is well established that nePTMs impair diverse protein functions, it can be expected that MGO-derived nePTMs of S100A12 may interfere with its RAGE-binding.

The aim of the present study was to test how MGO-derived nePTMs modulate RAGE binding. Furthermore, we were interested in the chemical modifications and molecular mechanisms that are responsible for the observed inhibition of receptor binding. Therefore, nePTM mapping was performed to identify the major molecular structures and binding sites of the predominant nePTMs. Analysis of the oligomerization behavior and defined synthesis and analysis of CEL-modifications of S100A12 led to a molecular model how MGO-derived nePTMs may reduce binding of S100A12 to RAGE.

## Materials and Methods

### Chemicals

IMPACT Kit, restriction enzymes, Crimson Taq DNA polymerase and T4 DNA ligase were purchased from New England Biolabs (Frankfurt am Main, Germany). The 2xYT-medium was obtained from Carl Roth (Karlsruhe, Germany). HBS-P+10x Buffer was supplied by GE Healthcare (Freiburg, Germany) and recombinant human RAGE Fc chimera from a mouse myeloma cell line by R&D Systems (Wiesbaden, Germany). Calibration standards for size exclusion chromatography, MGO (40% aqueous solution), sodium pyruvate, and liquid chromatography-mass spectrometry (LC-MS)-grade acetonitrile were purchased from Sigma-Aldrich (Taufkirchen, Germany). GluC and chymotrypsin were supplied by Roche (Mannheim, Germany). 2,5-Dihydroxyacetophenone (DHAP) and protein and peptide calibration standards for matrix-assisted laser desorption/ionization time-of-flight (MALDI-TOF)-MS were obtained from Bruker Daltonik (Bremen, Germany). All other chemicals were, unless otherwise noted, purchased from Sigma-Aldrich or Acros (Geel, Belgium) and were at least of analytical grade. H_2_O was purified water from a Synergi-185 laboratory water system (Millipore, Schwalbach, Germany).

### Construction of the expression plasmid

The cDNA for S100A12 was cloned into the expression vector pTXB1 using the IMPACT Kit. The expression of a fusion protein containing the target protein, an intein and a chitin-binding domain allowed the purification in a single step including the cleavage of the target protein from the intein [20]. I.M.A.G.E cDNA clone [21] containing the coding sequence for S100A12 (I.M.A.G.E cloneID 30415232) was obtained from imaGenes (Berlin, Germany). S100A12 was amplified by polymerase chain reaction (PCR) using the following oligonucleotides (Eurofins MWG, Ebersberg, Germany): 5′-GGTGGTCATATGACAAAACTTGAAGAG-3′ and 5′-GGTGGTACTAGTGCATCTCCCGTGATGCACTCTTTGTGGGTGTGG-3′. The restriction-digested PCR-product was ligated into the NdeI and SpeI site of pTXB1. The recombinant clones were confirmed by DNA sequencing. The resulting plasmid was transformed into *Escherichia coli* strain One Shot BL21 (DE3; Invitrogen, Darmstadt, Germany).

### Purification of recombinant S100A12

*Escherichia coli* cells were grown in 500 mL of 2xYT-medium containing 100 µg mL^−1^ of ampicillin at 37 °C and 150 rpm until the optical density (OD)_600_ reached 0.4. Protein expression was induced by adding isopropyl-β-d-thiogalactopyranoside (IPTG) to a final concentration of 400 µM. After incubation at 37 °C for 4 h, cells were harvested by centrifugation (4000 rpm, 4°C, 20 min). The cell pellet was re-suspended in 50 mL of ice-cold column buffer (20 mM Tris-HCl, 500 mM NaCl, 1 mM ethylenediaminetetraacetic acid (EDTA), pH 8.5) and lysed with a BeadBeater (Biospec, Bartlesville, OK). After centrifugation (11000 rpm, 4 °C, 30 min), the supernatant was loaded onto a chitin column containing 5 mL of chitin beads equilibrated with column buffer. The column was washed with 100 mL of column buffer and on-column cleavage was induced by flushing with 15 mL of cleavage buffer (column buffer containing 50 mM dithiothreitol (DTT)). Then the column flow was stopped and the column was incubated for 18 h at room temperature. S100A12 was eluted with 10 mL of column buffer. The eluate was dialyzed three times for 2 h against H_2_O, analyzed by sodium dodecyl sulfate polyacrylamide gel electrophoresis (SDS-PAGE) to confirm protein mass and purity and freeze-dried. S100A12 was dissolved in H_2_O and loaded onto an Illustra NAP-5 column (GE Healthcare, Solingen, Germany) equilibrated with H_2_O. After elution with H_2_O, S100A12 was freeze-dried. The amino acid sequence of S100A12 was confirmed with sequence coverage of 100% by MALDI-TOF-MS after digestion with GluC. The circular dichroism (CD) spectra of the protein showed proper folding of S100A12, the spectra being in accordance with literature (data not shown) [22]. Protein concentrations were determined with the Bio-Rad Protein Assay (Munich, Germany).

During cleavage of the target protein from the intein, one methionine residue preceeding the N-terminus was only partially cleaved, so that the signal for M1S100A12 was predominant in the S100A12 mass spectra. The N-terminal M residue, however, did not impair receptor binding, oligomerization, or MS analysis.

The present study investigated the influence of MGO-derived modifications on oligomerization and RAGE binding. Therefore, the functionality of the recombinant S100A12 was verified by size-exclusion chromatography and surface plasmon resonance spectroscopy (SPR; see below).

### Modification of S100A12 with MGO

Aliquots of 900 µL each of a S100A12 solution (2.6 mg mL^−1^ in 200 mM phosphate buffer, pH 7.4 with 0.04% sodium azide) were mixed with 100 µL each of 40 µM, 400 µM, 4 mM, and 40 mM MGO or 100 µL of buffer, respectively. The resulting solutions (final MGO concentrations were 0 µM, 4 µM, 40 µM, 400 µM, and 4 mM) were filter-sterilized (sterile syringe filter, 0.22 µm, PVDF, Carl Roth, Karlsruhe, Germany) and aliquots of 200 µL were incubated at 37 °C in a dry block shaker (350 rpm, Eppendorf, Hamburg, Germany) for 1, 3, and 7 days respectively. An unincubated sample without MGO served as negative control. Samples were stored at –20 °C until ultrafiltration.

### CEL-modification of S100A12

CEL-modified S100A12 was synthesized according to Koito et al. [23], with modifications. S100A12 was incubated with various concentrations of pyruvate and sodium cyanoborohydride to provide samples with different extents of modification. Briefly, 280 µL of a S100A12 solution (3.5 mg mL^−1^ in 200 mM phosphate buffer, pH 7.8) was mixed with 35 µL of NaBH_3_CN solution (0.17 mM, 0.33 mM, 1.67 mM, 8.35 mM, and 16.70 mM) and 35 µL of pyruvate solution (3 mM, 6 mM, 30 mM, 150 mM, and 300 mM) or 70 µL of buffer, respectively, and filter-sterilized. Aliquots of 200 µL were incubated at 25 °C and 350 rpm for 16 h. An unincubated sample without pyruvate and NaBH_3_CN served as negative control. Samples were stored at –20 °C until ultrafiltration.

### Removal of excess glycating reagent

Amicon Ultra-0.5 mL centrifugal filters were washed by centrifugation (14000 rpm, 30 min, 20 °C) of 200 µL of H_2_O. Samples were transferred to the filter, centrifuged and washed four times with 200 µL of water. The filter device was placed upside down in a new micro centrifuge tube and the protein solution was recovered by centrifugation (1000 rpm, 2 min, and 20 °C). The filter was washed once with 50 µL of H_2_O, which was combined with the recovered protein solution. Then the samples were freeze-dried and reconstituted with 200 µL of H_2_O.

### Investigation of the binding of S100A12 to RAGE by SPR

Twenty microliters of the samples was mixed with 50 µL of H_2_O, 10 µL of 5 mM CaCl_2_, 10 µL of 100 µM ZnCl_2_, and 10 µL of HBS-P+10x buffer. SPR measurements were performed on a Biacore X100 instrument (Biacore AB, GE Healthcare, Chalfont St. Giles, UK) at 25°C at a flow rate of 30 µL min^−1^. A quantity of 7200 response units (RU) of recombinant human RAGE Fc chimera was coupled to the measuring flow cell of a CM5 chip using amine coupling with *N*-hydroxysuccinimide/1-ethyl-3-(3-dimethylaminopropyl)carbodiimide according to the manufacturer’s instructions. Binding analysis was performed with HBS-P buffer (10 mM HEPES, 150 mM NaCl, 0.05% surfactant 20, pH 7.4) containing 500 µM CaCl_2_ and 10 µM ZnCl_2_ as running buffer. Samples were injected with a 180 s association and a 180 s dissociation phase to both flow cells. The response of the reference cell was subtracted from the response of the measuring cell to compensate for non-specific binding of S100A12 to the sensor chip. The sensor chip was regenerated with a 60 s pulse of 10 mM glycine, pH 2.0.

### Determination of the oligomerization behavior with size-exclusion chromatography

Aliquots of 30 µL each of the samples were mixed with 30 µL of H_2_O and 15 µL of buffer (50 mM Tris-HCl, 750 mM NaCl, 2.5 mM CaCl_2_, 50 µM ZnCl_2_, pH 7.4) and centrifuged at 20 °C and 10000×g for 10 min. Fifty microliters of the supernatant was injected into the HPLC system for analysis. The Jasco HPLC system (series 1580, Jasco Deutschland, Groß-Umstadt, Germany) consisted of a PU-1580 pump, a DG-1580-53 degasser, a LG-1580-02 ternary gradient unit, an AS-1555 autosampler and a MD-1510 diode array detector. A Superdex 75 10/300 GL column was used with 10 mM Tris-HCl, 150 mM NaCl, 500 µM CaCl_2_, 10 µM ZnCl_2_, pH 7.4 as running buffer and a flow rate of 0.6 mL min^−1^. The column was calibrated with bovine serum albumine, carbonic anhydrase, cytochrom C, and aprotinin for each test series. The chromatographic data were collected and processed using Borwin software.

### Analysis of nePTMs of S100A12 by MALDI-TOF-MS

For analysis of the intact protein, 10 µL of the samples was purified with ZipTip pipette tips (Millipore, Schwalbach, Germany) as described by Meltretter et al. [24]. Three microliters of the purified protein solution was mixed with 3 µL of 2% trifluoro acetic acid and 3 µL of DHAP matrix. An aliquot of 1 µL was spotted onto a ground steel target and air-dried. For the DHAP matrix, 7.6 mg of DHAP were suspended in 375 µL of ethanol and 125 µL of diammonium hydrogen citrate (80 µM) was added to dissolve the DHAP.

Peptides were analyzed after digestion with GluC. Therefore, 10 µL of the samples was mixed with 2.75 µL of 125 mM ammonium bicarbonate buffer, pH 8.0 and 1 µL of GluC (0.5 µg µL^−1^). Subsequently the mixture was incubated at 25 °C and 350 rpm for 16 h. A quantity of 3 µL of the digested protein was mixed with 3 µL of 2% trifluoro acetic acid and 3 µL of DHAP matrix; an aliquot of 1 µL was spotted onto a ground steel target and air-dried.

MALDI-TOF-MS analysis was carried out on a Bruker Autoflex system (Bruker Daltonik, Bremen, Germany) equipped with a nitrogen laser (λ = 337 nm). Measurements of intact and digested proteins were performed using delayed extraction (330 ns and 150 ns, respectively). Laser-desorbed positive ions were analyzed after acceleration by 20 kV in the linear mode for the intact proteins and by 19 kV in the reflector mode for the partial hydrolyzed protein. External calibration was conducted with a mix of insulin, ubiquitin I, cytochrome C, and myoglobin for the intact proteins and with a mix of bradykinin 1–7, angiotensin I and II, substance P, bombesin, renin substrate, ACTH clip 1–17 and 18–39, and somastostatin 28 for the peptides. For each spectrum, 150 laser shots were averaged from several positions on a spot. Data evaluation was performed with mMass [25].

### Analysis of nePTMs of S100A12 by UHPLC-ESI-MS/MS

MGO-modified S100A12 was analyzed after digestion with GluC or chymotrypsin, while CEL-modified S100A12 was digested only with GluC. Aliquots of 12 µL of the samples were mixed with 3.3 µL of 125 mM ammonium bicarbonate buffer, pH 8.0 and 1.2 µL of GluC (0.5 µg µL^−1^) or chymotrypsin (0.5 µg µL^−1^), respectively. For digestion with GluC, the solution was incubated at 37 °C and 350 rpm for 16 h. For digestion with chymotrypsin, the solution was incubated at 25 °C and 350 rpm for 16 h. After addition of 63.5 µL of H_2_O, 10 µL of acetonitrile, and 10 µL of 5% formic acid, the samples were filtered and aliquots of 10 µL each were injected into the ultrahigh-performance liquid chromatography (UHPLC)-electrospray ionization (ESI)-MS/MS system. An Ultimate 3000 RS UHPLC (degasser, binary pump, autosampler, column oven; Dionex, Germering, Germany) was coupled to an API 4000 QTRAP mass spectrometer equipped with an ESI-source (AB Sciex, Foster City, CA). Analyst 1.5.1 with BioAnalyst extensions was applied for instrument control as well as data acquisition and processing. An ACQUITY UPLC BEH300 C18 column (100 mm×2.1 mm, 1.7 µm particle size; Waters, Eschborn, Germany) was used at 30 °C with the following gradient: A, formic acid (0.1%); B, acetonitrile; flow rate, 0.3 mL min^−1^; (time [min]/% B) −5.0/5, 0.0/5, 5.0/5, 55.0/50, 55.5/95, 60.0/95. All flow eluting before 2.0 or after 40.0 min was discarded by a two-position valve prior to mass analysis. The ESI-source was operated at 500 °C with a voltage of +5500 V and nitrogen as drying gas. Time periods were chosen to perform different product ion scans during one chromatographic run. Declustering potential, collision energy and scanned mass range were optimized for each peptide.

## Results

### MGO induces impaired binding of S100A12 to RAGE

**Figure 1:**
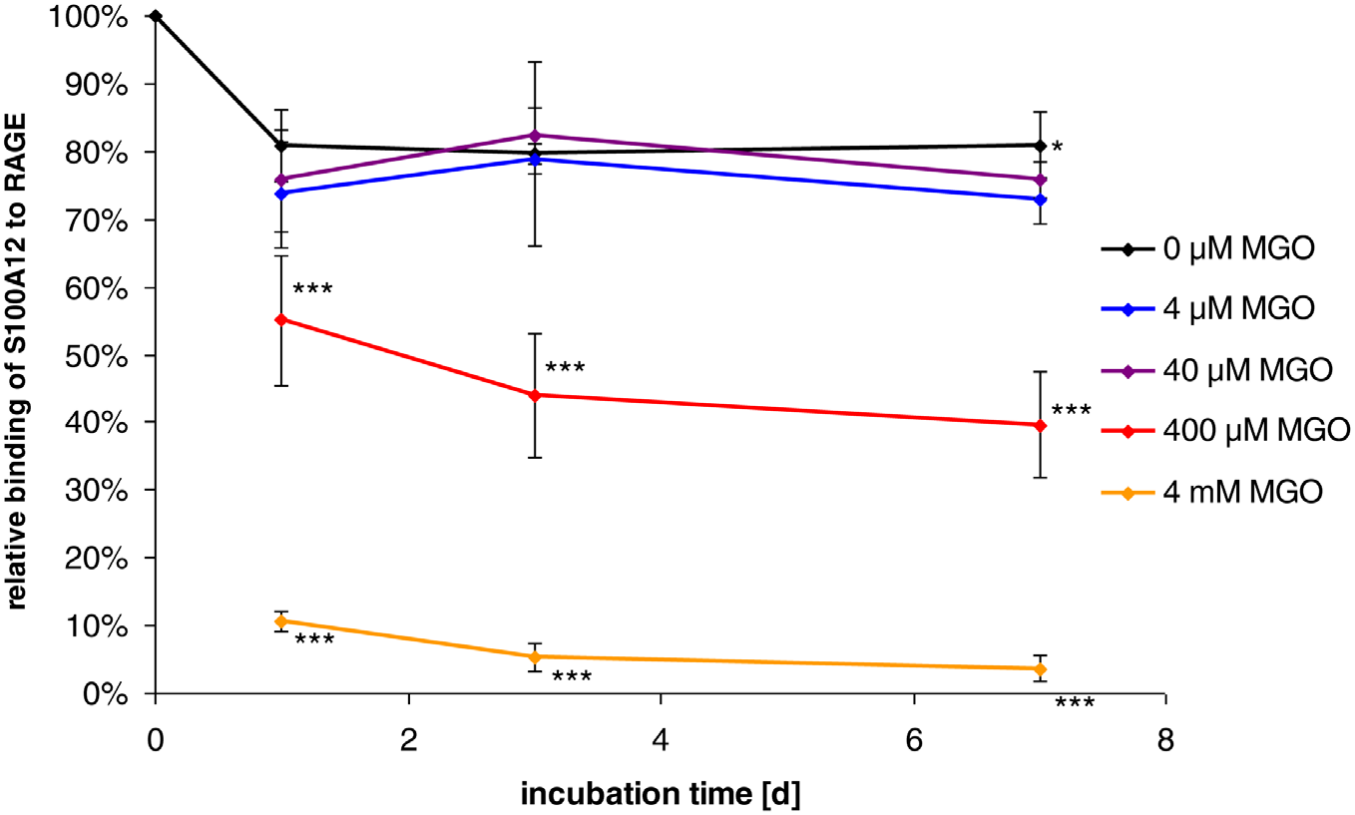
Effects of methylglyoxal (MGO) on the binding of S100A12 to the receptor of advanced end-products (RAGE). S100A12 was incubated with different concentrations of MGO at 37 °C for 1, 3, and 7 days. Binding of the modified S100A12 to RAGE was investigated with surface plasmon resonance spectroscopy. The binding of the unincubated negative control was set as 100%. Values represent mean ± SD of three independent experiments with duplicate measurements; *p<0.05, ***p<0.001, significant differences are related to the control (0 µM MGO).

The present study investigated the effect of MGO-induced nePTM on the structure and functionality of S100A12. For this purpose, S100A12 was incubated with different concentrations of MGO (4 µM, 40 µM, 400 µM, and 4 mM) for different periods of time (1, 3, and 7 days) and binding of S100A12 to RAGE was analyzed by SPR spectroscopy. The physiologic running buffer (HBS-P) was supplemented with 500 µM Ca^2+^ and 10 µM Zn^2+^, because S100A12 interacts with RAGE dependent on these metal ions [13]. The applied concentrations of Ca^2+^ and Zn^2+^ were in the range of their serum levels. The recombinant RAGE was derived from a mouse myeloma cell line. The cell source may have major influence on the functionality of RAGE. Recently, it was shown that soluble RAGE (sRAGE) from mammalian cells exerts higher bioactivity than sRAGE from insect cells or bacterial cells [26]. Most likely, carboxylated N-glycans on sRAGE, which are formed only in mammalian cells, promote binding to S100 proteins [27]. (Figure 1) shows the results of the binding analysis with SPR. The maximum response was observed for the unincubated negative control with a value of 3047 RU.

The negative control incubated without MGO showed a slight decrease in binding to 80.9±5.1% (mean ± SD) after seven days compared to the unincubated control indicating that the incubation itself had an influence on the binding of S100A12 to RAGE. For this reason, samples were compared to the negative control incubated for the same period of time, but without MGO. The samples incubated with 4 µM and 40 µM MGO exhibited a similar binding behavior as the negative control. The incubation with 400 µM MGO led to a reduction of binding to 55.0±9.6% after one day with further decrease during prolonged incubation. The reaction with 4 mM MGO reduced the binding to 10.7±1.5% after one day with a further decline over the incubation time to 3.7±1.8% after seven days.

### Influence of MGO on S100A12 oligomerization

**Figure 2:**
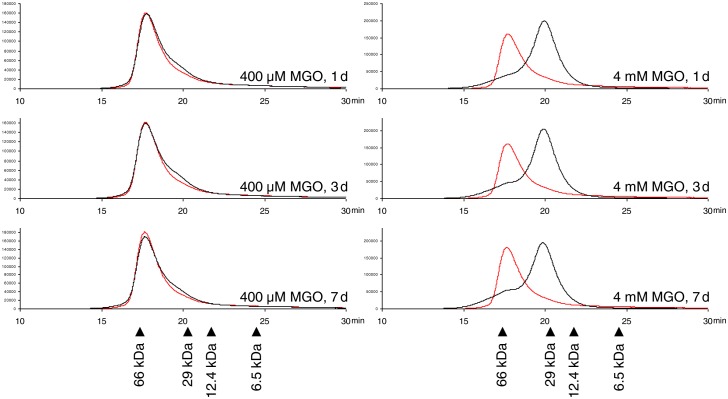
Concentration-dependent effect of methylglyoxal (MGO) on the ability of S100A12 to form hexamers. S100A12 was incubated with different concentrations of MGO at 37 °C for 1, 3, and 7 days. Oligomerization behavior was analyzed with size-exclusion chromatography. The negative control incubated for the same time without MGO is shown in red. Chromatograms are representative for two independent experiments.

Because the active form of S100A12, which binds to RAGE, is a hexamer, the influence of MGO on the hexamerization of S100A12 was investigated by size-exclusion chromatography. A physiologic running buffer with 500 µM CaCl_2_ and 10 µM ZnCl_2_ was used, because the formation of S100A12 hexamers is also dependent on Ca^2+^ and Zn^2+^ [13]. The chromatograms of the negative controls (overlay in red) showed one peak with a retention time of 17.6 min (Figure 2). This retention time corresponds to the molecular weight of about 57 kDa, which is in the size range of the S100A12 hexamer indicating that the hexamer was actually formed under the applied conditions, and that the incubation itself had no influence on the hexamerization. The samples modified with 4 µM and 40 µM MGO showed the same elution profile (data not shown) indicating that the formation of the hexamer was not impaired under the reaction conditions. The peaks resulting from the samples modified with 400 µM MGO exhibited a small shoulder towards smaller molecular weight suggesting that a minor part of S100A12 was not present as hexamer. The effect, however, was independent from the incubation time suggesting a rapid reaction of MGO with the protein. The chromatograms of the samples incubated with 4 mM MGO, in contrast, displayed only a small part of the molecules present as hexamers, whereas the main peak had a retention time of 19.9 min corresponding to a molecular weight of 28 kDa. This molecular weight is in the range of a trimer. Because no trimer of S100A12 has been reported before, the peak may also represent equilibrium between the S100A12 dimer and the tetramer. Although the slope of the calibration curve was relatively high (1 kDa 0.1 min^−1^), it could also be possible that the signal represented the dimer, if the accuracy of the method was low or if the dimer showed inconsistent retention behavior. Thus, the high modification rate caused by 4 mM MGO resulted in a major impairment of S100A12 hexamerization.

### Mass spectrometric nePTM mapping of MGO-modified S100A12

To investigate the molecular determinants of the reduced binding of MGO-modified S100A12 to RAGE and of the impaired hexamerization, the structures and binding sites of the resulting nePTMs were analyzed by MS. In the first step, untargeted analysis of nePTMs was performed by MALDI-TOF-MS of the intact S100A12 to gather a profile of all major structures that had been formed. In the second step targeted nePTM analysis was performed by UHPLC-ESI-MS/MS to identify the binding sites. MALDI-TOF-MS allows the untargeted analysis of the present nePTM modifications, because all major modifications appear as satellite signals of the native protein in the MALDI spectrum, independent from the structure of the modification [28]. The structures of the modifications were assigned by the mass difference between MGO-modified and the unmodified protein (Table 1) using data from literature [2], [29]–[31].

**Table 1:** Overview of detected modifications of S100A12 after incubation with methylglyoxal at 37 °C.

Mass shift (Da)				
4 µM MGO	40 µM MGO	400 µM MGO	4 mM MGO	Interpretation
		+54	+54	Hydroimidazolone
		+72		CEL/MGO-hemiaminals/dihydroxyimidazolidine/CEA
			+80	Argpyrimidine
			+126	Dihydropyrimidine/hydroimidazolone + CEL (54+72 Da)
			+144	Tetrahydropyrimidine/CEL + hemiaminals (72+72 Da)/2×CEL (72+72 Da)

The intact protein was analyzed by MALDI-TOF-MS. All modifications were detected after one, three, and seven days of incubation. The table shows the results of three independent experiments; MGO, methylglyoxal; CEL, *N^ε^*-carboxyethyllysine; CEA, carboxyethylarginine.

**Figure 3:**
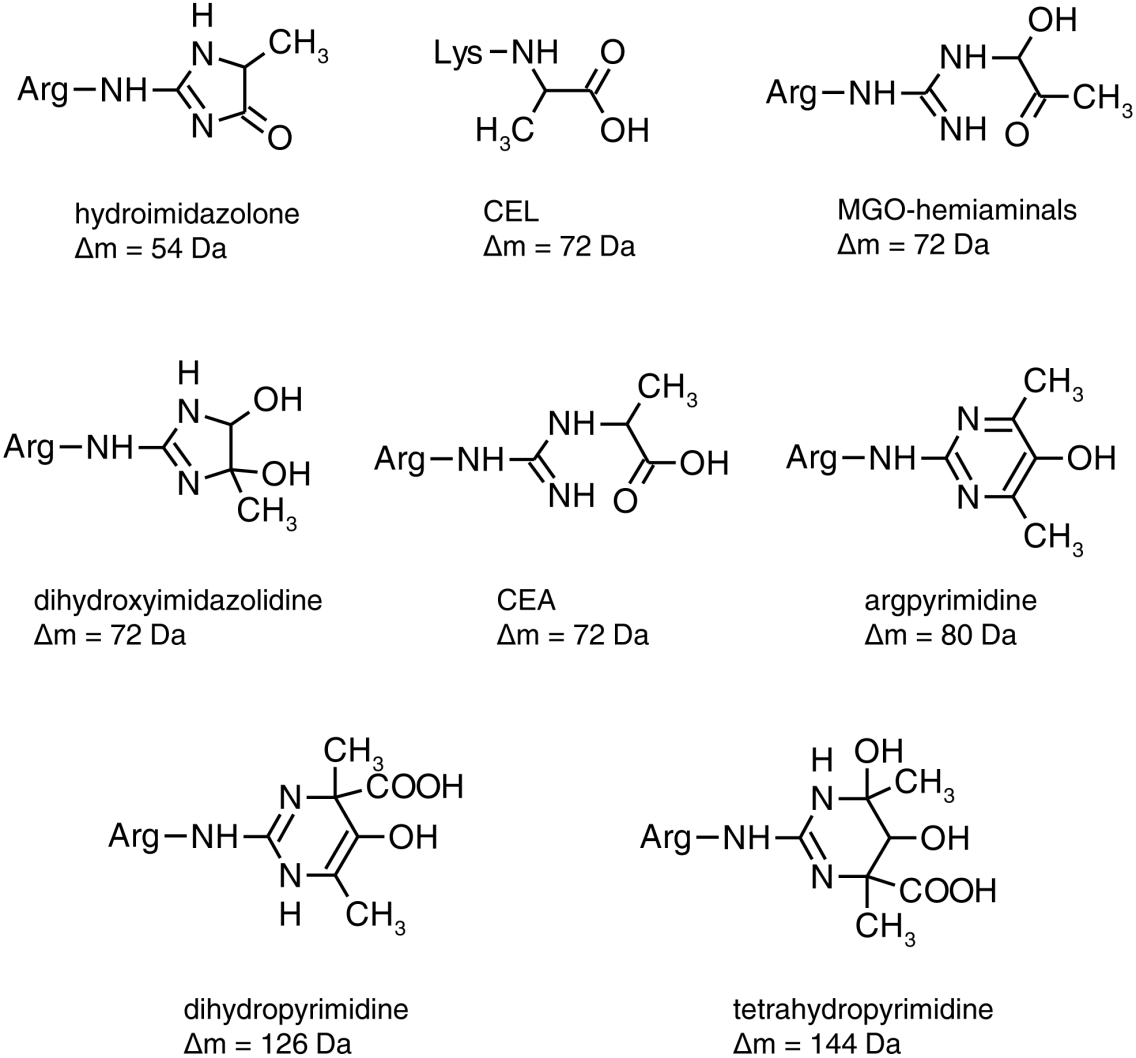
Structures of different advanced glycation end-products and their corresponding mass increase.

Two additional peaks were detected in the samples incubated with 400 µM MGO for one day. One peak showed a mass increase of +54 Da related to the native protein, which corresponds to the arginine modification hydroimidazolone (Figure 3). The other peak displayed a mass shift of +72 Da, which could be caused by several modifications on the arginine or lysine side chains. Arginine could react with MGO to hemiaminals (MGO-hemiaminals or dihydroxyimidazolidine) or to carboxyethylarginine (CEA), which all have the same molecular weight. Alternatively, lysine could react to CEL, which causes the same mass increase. After the incubation with 4 mM MGO for one day, no signal of the native protein could be discerned suggesting that it was modified to a high extent. The hydroimidazolone was detected, but there was no peak indicating the +72 Da-modification. Instead, there were three additional peaks with a mass shift of +80 Da, +126 Da, and +144 Da, respectively. The mass increase of +80 Da can be explained by the arginine modification argpyrimidine, while the mass shift of +126 Da could be assigned to dihydropyrimidine formed on the arginine side chain or to a concurrent modification of the arginine residue to the hydroimidazolone (+54 Da) and of the lysine residue to CEL (+72 Da). There are also two possible explanations for the +144 Da-modification, which could be the arginine modification tetrahydropyrimidine or a twofold modification with CEL. Untargeted MALDI-TOF-MS analysis was also repeated after partial enzymatic hydrolysis with GluC to increase the sensitivity (data not shown). After hydrolysis, the modifications were observed already at lower MGO concentrations, but no additional MGO-derived nePTM structures were detected.

For the identification of binding sites of the protein modifications in the amino acid sequence, the modified protein S100A12 was analyzed by targeted UHPLC-ESI-MS/MS after partial hydrolysis with the endoproteinase GluC. On the one hand, the localization of the binding site facilitates the prediction how a modification contributes to the observed loss of function. On the other hand, verification of ambiguous structure assignments could be achieved. Finally, targeted UHPLC-ESI-MS/MS analysis is much more sensitive than MALDI-TOF-MS, so that modifications of proteins incubated with lower MGO-concentrations could also be analyzed.

For this purpose, product ion scans of all possible modified peptides were performed. Thus, the *m/z*-values of the peptides containing lysine and/or arginine residues with all possible modifications detected by untargeted MS analysis were calculated. Subsequently, product ion scans were performed using the calculated *m/z*-values as precursor ions. The modification sites were identified by fragment patterns showing the mass shift of the modification [32]. The peptide AA 10–32 fragmented very poorly, so that the amino acids of interest (R21, K22, and K30) were investigated in peptides AA 19–28 and AA 29–33 after digestion with chymotrypsin.

Table 2 depicts the detected modifications and their assignment to the modified amino acid. After incubation with 4 µM MGO, only hydroimidazolone with a mass shift of +54 Da and a modification with a mass shift of +72 Da were detected, both at R21. Thus, it was confirmed that the +72 Da-modification was located on the arginine and was not caused by CEL. As mentioned above, a +72 Da-modification at an arginine side chain could be due to hemiaminals or CEA. However, the product ion spectra of the peptide R_21+72 Da_ showed a loss of water at many fragments as well as a loss of the whole modification at some fragments. This fragmentation behavior strongly indicates that the peptide was modified by hemiaminals, which are relatively loosely bound early reaction products, and not by CEA [2]. This assignment is also supported by the time course of the formation of peptide R_21+72 Da_: The peak area of this modified peptide grew with increasing MGO concentration until 400 µM, but the peak areas of the samples modified with 4 mM MGO were about 10-fold smaller than the peak areas of the samples modified with 400 µM MGO and were similar to the peak areas of the samples modified with 40 µM MGO. This behavior indicates that the modification is a reaction product which undergoes further reactions with MGO, confirming the presence of a relatively labile intermediate such as a hemiaminal. However, the presence of CEA cannot be fully excluded.

**Table 2:** Overview of detected modifications of S100A12 after incubation with methylglyoxal at 37°C for 1, 3, and 7 days.

		Mass shift (Da)					
Peptide	Amino acid sequence	4 µM MGO	40 µM MGO	400 µM MGO	4 mM MGO	Modification at	Interpretation
AA 1–6	MTKLEE		+72^a^	+72	+72	N-terminus	Carboxyethylation
			+72^a^	+72	+72	K3	CEL
AA 2–6	TKLEE			+72	+72	N-terminus	Carboxyethylation
				+72	+72	K3	CEL
AA 2–9	TKLEEHLE				+72	N-terminus	Carboxyethylation
					+72^a^	K3	CEL
AA 19–28	SVRKGHFDTL	+54	+54	+54	+54	R21	Hydroimidazolone
		+72	+72	+72	+72	R21	MGO-hemiaminal/CEA
			+80	+80	+80	R21	Argpyrimidine
			+144	+144	+144	R21	Tetrahydropyrimidine^b^
AA 29–33	SKGEL		+72^c^	+72	+72	K30	CEL
AA 33–40	LKQLLTKE		+72	+72	+72	K34	CEL
			+72	+72	+72	K39	CEL
AA 41–50	LANTIKNIKD			+72	+72	K46	CEL
				+72	+72	K49	CEL
AA 51–56	KAVIDE			+72^a^	+72	K51	CEL
AA 41–56	LANTIKNIKDKAVIDE			+72	+72	K46	CEL
			+72	+72	+72	K49	CEL
				+72	+72	K51	CEL
AA 74–92	FISLVAIALKAAHYHTHKE		+72	+72	+72	K83	CEL
			+72	+72	+72	K91	CEL

Peptides were analyzed with ultrahigh-performance liquid chromatography-electrospray ionization-tandem mass spectrometry after partial hydrolysis with GluC or chymotrypsin. Modifications were detected after one day of incubation with the exceptions as indicated in footnotes (a) and (c). The table shows the results of three independent experiments; *^a^* detected after three days, *^b^* five different peaks, *^c^* detected after seven days; MGO, methylglyoxal; CEL, *N^ε^*-carboxyethyllysine; CEA, carboxyethylarginine.

Incubation with 40 µM MGO additionally led to detectable levels of argpyrimidine and to tetrahydropyrimidine at R21. Five peaks with the mass of the peptide containing tetrahydropyrimidine were most likely caused by different isomers [33]. The peak areas of the argpyrimidine-modified peptide increased with both MGO concentration and incubation time indicating that argpyrimidine represents a stable end-product of the reaction of R21 with MGO. This observation is consistent with the study of Kloepfer et al. [29]. The modification with a mass increase of +126 Da, which was detected by MALDI-TOF-MS, could not be assigned to distinct amino acids by product ion spectra. Thus, it could not be elucidated if the mass increase represents the arginine modification dihydropyrimidine or a concurrent modification of the arginine residue to the hydroimidazolone and of the lysine residue to CEL. However, the assignment to dihydropyrimidine is more likely, because the chromatogram showed five peaks with the respective mass indicating a modification that can form different isomers.

CEL was detected in samples with 40 µM MGO at K3, K30, K34, K39, K49, K83, and K91. After incubation with 400 µM MGO, CEL was also identified at K46 and K51. Additionally, carboxyethylation of the N-terminus was observed.

### Analysis of CEL-modified S100A12

nePTM mapping of MGO-modified S100A12 revealed the presence of CEL at all lysine side chains as well as the presence of hydroimidazolone, argpyrimidine, tetrahydropyrimidine, and hemiaminals/CEA at the arginine residue R21. In order to investigate the potential role of the different nePTMs on hexamerization and RAGE-binding of S100A12, an S100A12 derivative was synthesized, which contained only CEL-modifications at all lysine residues, but no arginine modifications. For this purpose, reductive amination was carried out using different concentrations of pyruvate (300 µM, 600 µM, 3 mM, 15 mM, and 30 mM) and NaBH_3_CN.

The existence of CEL and the absence of other modifications were confirmed by targeted UHPLC-ESI-MS/MS analysis of CEL-S100A12 samples after digestion with GluC. Analysis revealed that all lysine residues of S100A12 carried the CEL-modification and that the N-terminus was also carboxyethylated. The respective modifications were present already at the lowest concentration of pyruvate (300 µM). The peak areas of the modified peptides in the chromatograms grew with increasing concentration of pyruvate indicating a rising modification rate. Comparison of the peak areas of CEL-modified and MGO-modified peptides revealed that all MGO-modified S100A12 samples had equal or lower CEL-modification rates than the CEL-S100A12 sample prepared with 3 mM pyruvate.

**Figure 4:**
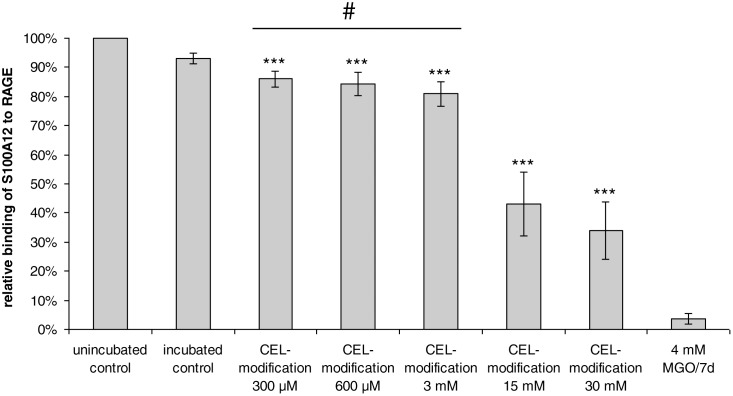
Effect of *N^ε^*-carboxyethyllysine (CEL)-modifications on the binding of S100A12 to the receptor for advanced glycation end-products (RAGE). S100A12 was incubated with the indicated concentrations of pyruvate and sodium cyanoborohydride at 25 °C for 16 h to achieve a similar CEL-modification as present in methylglyoxal (MGO)-modified S100A12. Binding of the CEL-modified S100A12 to RAGE was investigated with surface plasmon resonance spectroscopy. The binding of the unincubated negative control was set as 100%. #, in all MGO-modified proteins, CEL modification rates were ≤ those of CEL-S100A12 at 3 mM. Values represent mean ± SD of three independent experiments with duplicate measurements; ***p<0.001, significant differences are related to the incubated control. The CEL-modifications did not inhibit the hexamerization of S100A12 because there was no elution behavior towards smaller molecular weight compared to the negative control (data not shown).

To analyze the effect of the CEL-modifications on the binding of S100A12 to RAGE, the binding was investigated with SPR (Figure 4). The incubated control without pyruvate and sodium cyanoborohydride exhibited a binding rate of 93.2±1.8% compared to the unincubated control showing that the incubation had a small effect on the binding of S100A12 to RAGE. The binding of CEL-modified S100A12 to RAGE decreased with increasing modification rate. CEL-S100A12 prepared with 300 µM pyruvate showed a significantly reduced RAGE binding of 86.1±2.7% of the control (p<0.001). Using 600 µM, 3 mM, 15 mM, and 30 mM pyruvate, the binding of the resulting CEL-S100A12 decreased to 84.4±3.9%, 80.9±3.3%, 43.1±11.1%, and 34.0±9.7%, respectively.

## Discussion

The incubation of S100A12 with MGO led to different modifications dependent on the MGO concentration. At the lowest MGO concentration (4 µM), only hydroimidazolone and hemiaminals were detected at R21. Using 40 µM MGO, argpyrimidine and tetrahydropyrimidine were detected at R21 as well as CEL at four different lysine residues. At higher MGO concentrations, an additional CEL modification of most lysine residues was observed. These results confirm the higher reactivity of MGO towards arginine compared to lysine [34]. No other lysine modification besides CEL, a modification that is formed specifically between MGO and lysine [30], could be detected.

The tandem mass spectrometric analysis of the MGO-modified S100A12 revealed that almost all lysines and the N-terminus were carboxyethylated after incubation with 40 µM MGO or 400 µM MGO. As an exception, no CEL was detected at K22. Thus, K22 may be shielded against reaction with MGO due to early and readily formed adducts at the neighboring R21. Alternatively, CEL at K22 may not be detected under the applied conditions, e.g. due to poor ionization properties.

**Figure 5:**
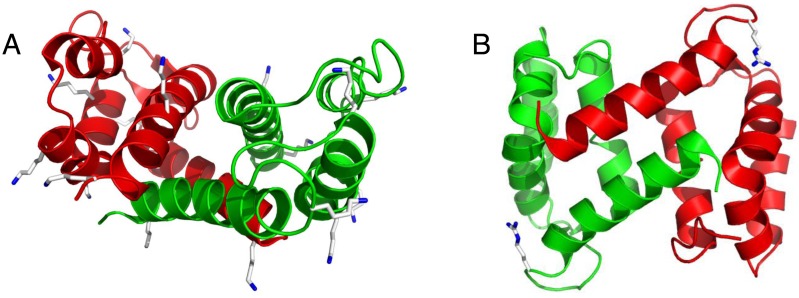
Localization of resolved lysine (A) or arginine (B) side chains in the crystal structure of S100A12 (PDB-Code 2WCB). The two subunits of the S100A12 dimer are shown in red and green. The C- and N-atoms of lysine or arginine side chains are colored white and blue, respectively.

The CEL-modified S100A12 carried CEL on all lysine residues even after incubation with the lowest concentration of pyruvate (300 µM). Hence, there was no indication of a site-specific modification of the lysine residues in S100A12 either by reductive amination or by MGO. In S100A12, all lysine residues are located on the surface of the dimer (Figure 5A) indicating that the lysine residues are easily and equally accessible for the glycation reagents. Preferential glycation, however, is dependent on the differing accessibility of lysine side chains [35]. The arginine side chains are also located on the surface of the dimer (Figure 5B) providing easy access for MGO. The good accessibility of the lysine and arginine side chains on S100A12 apparently prevails over other effects that could lead to site-specific modification. In contrast, site-specific modifications were suggested for proteins like human serum albumin [7], hemoglobin [36], or RNase [37].

In contrast to the CEL-modification of S100A12, the incubation with MGO affected the hexamerization of S100A12. The hexamer was formed after incubation with 4, 40, and 400 µM MGO. By contrast, the incubation with 4 mM MGO did not lead to an oligomerization exceeding the tetramer. The peak areas of the CEL-modified peptides prepared with 3 mM pyruvate were equal to or higher than the peak areas obtained for all MGO-modified S100A12. This result suggests that the CEL-modifications of lysine residues do not influence the formation of the S100A12 hexamer. Since MGO-modified S100A12 impairs hexamerization, it can be concluded that these deteriorations are caused by the detected modifications at R21, which are the only structural difference between MGO-S100A12 and CEL-S100A12.

**Figure 6:**
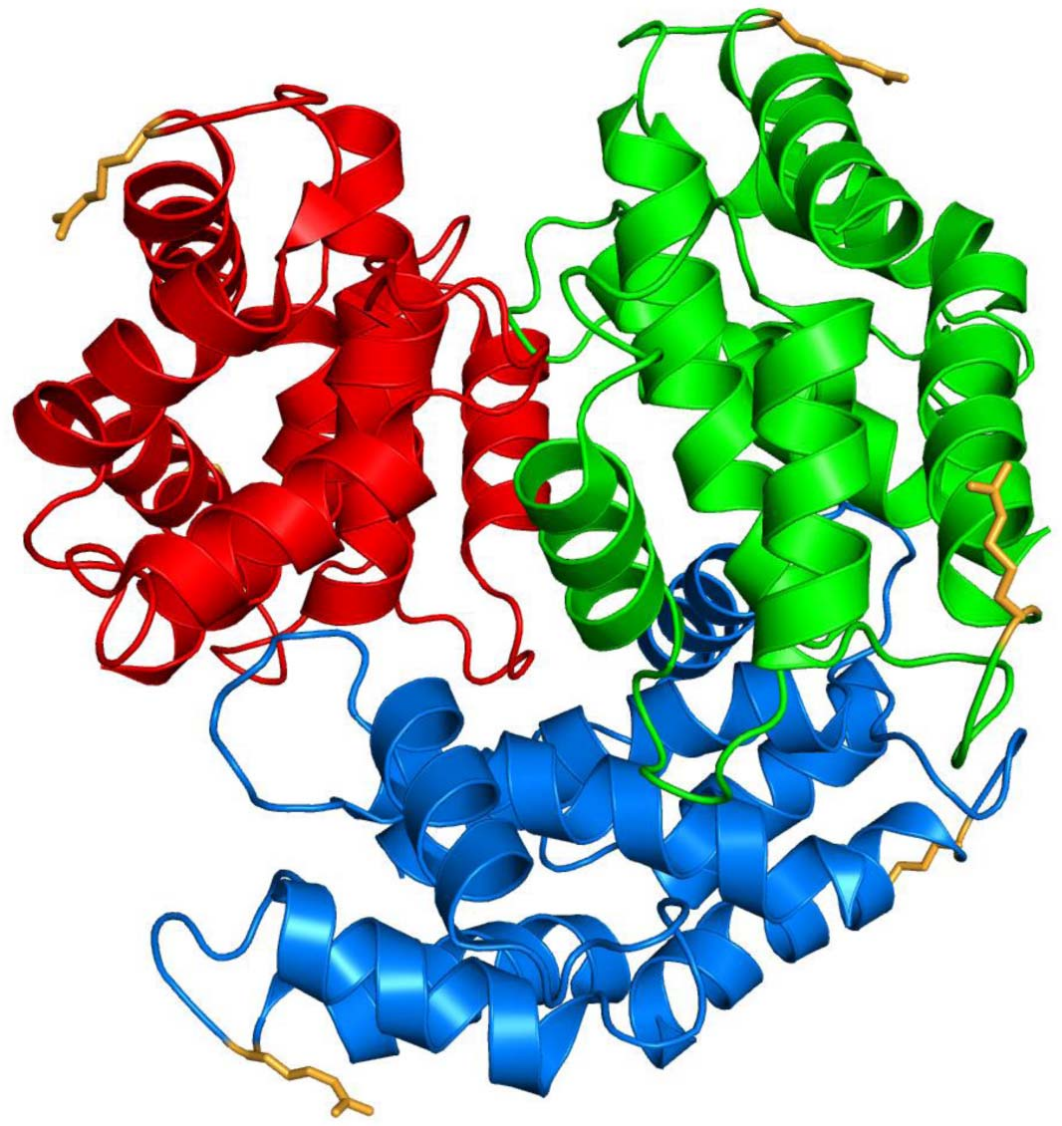
Localization of arginine side chains (orange) in the S100A12 hexamer (PDB-Code 1GQM). The three dimers of the S100A12 hexamer are shown in red, green, and blue, respectively.

**Figure 7:**
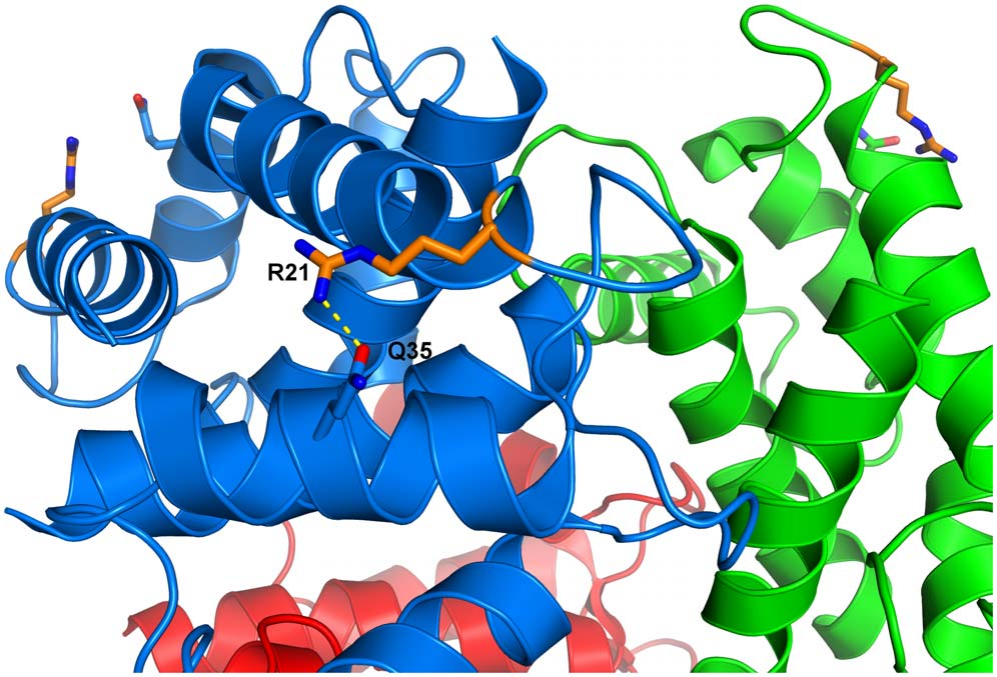
Environment of R21 in the S100A12 hexamer (PDBCode 2WCE). The three dimers are shown in blue, red, and green. Nitrogen and oxygen atoms are shown in blue and red, respectively.

The arginine side chains are located far from the hexamerization interface indicating that the inhibition of the hexamerization was not caused by a steric effect of the arginine modifications (Figure 6). One reason why R21 is important for hexamerization could be a stabilizing effect on the structure of S100A12. The guanidinium group of R21 is close enough to the amide group of Q35 to form a hydrogen bond (Figure 7). Q35 is located in an α-helix so that the hydrogen bond could stabilize the position of this α-helix relative to the loop containing the arginine. Modification of the arginine side chain could lead to a loss of this hydrogen bond causing an alteration of the tertiary structure of S100A12. This conformational change could then be the reason for impaired hexamerization.

SPR analysis revealed that the binding of S100A12 to RAGE was reduced after incubation with MGO in a concentration-dependent manner. The incubation of S100A12 with 4 µM and 40 µM MGO had no effect on its binding to RAGE consistent with the low modification degree detected by UHPLC-ESI-MS/MS. With increasing MGO concentration, the binding of S100A12 to RAGE declined consistently with the detection of more modifications.

The specific introduction of CEL into S100A12 led to a concentration-dependent decrease of the binding of S100A12 to RAGE. This result suggests that the binding declines with increasing modification rate. However, the lysine modification rate of CEL-S100A12 prepared with 3 mM pyruvate was equal to or higher than the CEL-modification rate of S100A12 incubated with 4 mM MGO. Relative binding of the former CEL-S100A12 to RAGE, in contrast, reached 81%, whereas RAGE binding of the latter MGO-modified S100A12 was down to 4%. These results indicate that the modifications of R21 by MGO are major factors leading to the functional loss of S100A12, whereas CEL-modifications at the lysine residues play only a minor role herein. It has been shown before for other proteins, such as small heat shock proteins or human serum albumin, that specific arginine residues can be important determinants for protein biofunction [7], [38]. The structural reason for the impairment of S100A12-binding to RAGE by the modification of R21 is not clear. No major impairment of hexamerization was recorded when MGO concentrations lower than 4 mM were used, whereas even the incubation with 400 µM led to more than 50% suppression of RAGE binding. Therefore, it can be concluded that impaired S100A12 oligomerization is not the only mechanism leading to MGO-induced suppression of RAGE binding. Similar to the dimer, R21 is located on the surface of the S100A12 hexamer, which is the active form of the ligand (Figure 6). Thus, it is possible that the reduced binding as a consequence of R21 modification is due to steric interference at the S100A12–RAGE binding interface.

Xie et al. suggested that the formation of a Ca^2+^-complex of S100A12 is a prerequisite of RAGE binding that leads to drastic conformational changes which allow interaction of Ca^2+^-S100A12 with RAGE [39]. Because calcium is coordinated, besides others, by K22, the modification of the neighboring R21 may impair calcium-coordination and subsequent conformational changes necessary for receptor binding. Furthermore, MGO-induced modification of R21 may additionally deteriorate the RAGE-binding site of S100A12. However, the structural basis of the RAGE–S100A12 interaction is not fully understood. Xie et al. described binding sites of the S100A12 hexamer that mediate binding to the C1C2 domain of tetramer RAGE [39]. In contrast, Dattilo et al. postulated that binding of S100 ligands is rather directed towards the V-domain of RAGE with secondary effects of the C1 domain [40]. Consequently, binding mechanisms of S100B to the VC1 domain of RAGE were described [41]. However, these results cannot be directly transferred to S100A12, because different S100 proteins are assumed to bind to different sites of RAGE [42]. Therefore, further studies on the structural basis of RAGE–S100A12 interaction are required to determine the mechanism how MGO-induced modifications of the ligand may directly interfere with receptor binding.

The formation of nePTMs may modulate RAGE signaling by different mechanisms. It is well established that CML-modifications convert non-ligands into strong agonists of RAGE [15], [19]. Since CML-modified S100A8 und S100 A8/9 were detected in inflamed gut tissue, it can be speculated that CML-modifications further enhance the binding of these natural ligands of RAGE [19]. In the present study, MGO-induced nePTMS, mainly hydroimidazolone, inhibited RAGE binding. Because hydroimidazolone is present *in vivo* in higher concentrations than CML [3], it can be hypothesized that the inhibitory effect of hydroimidazolone overcompensates a possible advancing effect of CML.

Geczy and coworkers found that the S100 proteins S100A8 and S100A9 are susceptible to oxidative modifications *in vivo* leading to alterations of the proinflammatory function [43]. Thus, they proposed that pronounced oxidation of S100A8 and S100A9, which is observed during inflammation, may act as a regulatory switch suppressing excessive inflammation. However, it remains speculation if the inactivating modification of S100A12 by MGO exerts a similar regulatory effect restoring homeostasis as the oxidation of S100A8 and S100A9.

## Conclusion

It has been shown that S100 protein is subjected to nePTM *in vivo* at inflammation sites, which may influence its binding to the natural receptor RAGE [19]. In the present study, mechanisms were investigated how nePTMs may modulate S100A12–RAGE binding. Thus, nePTMs were introduced into S100A12 by MGO, which is a major nePTM precursor *in vivo* [44]. The formation of MGO-derived nePTMs led to a time- and concentration-dependent decrease of RAGE–S100A12 binding, which is accompanied by an interference of S100A12 hexamerization at higher MGO concentrations. MS analysis revealed the presence of CEL-modifications at nine of the lysine residues and the N-terminus as well as at least four different modifications at R21. Synthetically prepared CEL-modified S100A12 also carried CEL-residues at all lysine residues and the N-terminus in concentrations as present in MGO-S100A12, but showed only minor impairment of RAGE-binding and no impairment of hexamerization. Thus, it can be concluded that mainly modifications at R21 are responsible for the functional consequences of S100A12-nePTMs. Most likely, reduced binding to RAGE is caused -to a minor extent- by allosteric inhibition of S100A12 hexamerization and mainly by allosteric or steric interference of the ligand-receptor interface.
